# Implementation of a Diabetes Self-Management Education and Support Intervention in Rural Guatemala: A Mixed-Methods Evaluation Using the RE-AIM Framework

**DOI:** 10.5888/pcd18.210259

**Published:** 2021-12-09

**Authors:** Scott Tschida, David Flood, Magdalena Guarchaj, Juanita Milian, Andrea Aguilar, Meredith P. Fort, Timothy Guetterman, Carlos Mendoza Montano, Ann Miller, Lidia Morales, Peter Rohloff

**Affiliations:** 1 Center for Research in Indigenous Health, Wuqu’ Kawoq, Tecpán, Chimaltenango, Guatemala; 2 Department of Internal Medicine, National Clinician Scholars Program, University of Michigan, Ann Arbor, Michigan; 3 Instituto de Salud Incluyente, San Lucas Sacatepéquez, Sacatepéquez, Guatemala; 4 Colorado School of Public Health, University of Colorado Anschutz Medical Campus, Aurora, Colorado; 5 Department of Family Medicine, University of Michigan, Ann Arbor, Michigan; 6 Centro de Investigación para la Prevención de las Enfermedades Crónicas, Instituto de Nutrición de Centro América y Panamá, Guatemala City, Guatemala; 7 Department of Global Health and Social Medicine, Harvard Medical School, Boston, Massachusetts; 8 Division of Global Health Equity, Brigham and Women’s Hospital, Boston, Massachusetts

## Abstract

**Introduction:**

To address the global diabetes epidemic, lifestyle counseling on diet, physical activity, and weight loss is essential. This study assessed the implementation of a diabetes self-management education and support (DSMES) intervention using a mixed-methods evaluation framework.

**Methods:**

We implemented a culturally adapted, home-based DSMES intervention in rural Indigenous Maya towns in Guatemala from 2018 through 2020. We used a pretest–posttest design and a mixed-methods evaluation approach guided by the RE-AIM (Reach, Effectiveness, Adoption, Implementation, Maintenance) framework. Quantitative data included baseline characteristics, implementation metrics, effectiveness outcomes, and costs. Qualitative data consisted of semistructured interviews with 3 groups of stakeholders.

**Results:**

Of 738 participants screened, 627 participants were enrolled, and 478 participants completed the study. Adjusted mean change in glycated hemoglobin A_1c_ was −0.4% (95% CI, −0.6% to −0.3%; *P* < .001), change in systolic blood pressure was −5.0 mm Hg (95% CI, −6.4 to −3.7 mm Hg; *P* < .001), change in diastolic blood pressure was −2.6 mm Hg (95% CI, −3.4 to −1.9 mm Hg; *P* < .001), and change in body mass index was 0.5 (95% CI, 0.3 to 0.6; *P* < .001). We observed improvements in diabetes knowledge, distress, and most self-care activities. Key implementation factors included 1) recruitment barriers for men, 2) importance of patient-centered care, 3) role of research staff in catalyzing health worker involvement, 4) tradeoffs between home and telephone visits, and 5) sustainability challenges.

**Conclusion:**

A community health worker–led DSMES intervention was successfully implemented in the public health system in rural Guatemala and resulted in significant improvements in most clinical and psychometric outcomes. Scaling up sustainable DSMES in health systems in rural settings requires careful consideration of local barriers and facilitators.

**Summary**

**What is already known on this topic?**

The burden of diabetes is large and growing in low- and middle-income countries. A significant gap exists in how to optimally incorporate lifestyle counseling interventions into health systems in these countries.

**What is added by this report?**

We assessed implementation of a large diabetes self-management education and support (DSMES) program in rural Guatemala. This report highlights information on implementation barriers and facilitators that will be useful to implementers and policy makers who work to scale up DSMES in resource-limited health systems.

**What are the implications for public health practice?**

Rigorous DSMES interventions can be successfully implemented in rural public health systems in low- and middle-income countries, although challenges include enrollment of men, additional work for overburdened health workers, and sustainability.

## Introduction

The number of adults with diabetes is estimated to grow worldwide from 463 million in 2019 to 700 million in 2045 (1). More than 80% of the diabetes burden is in low- and middle-income countries (2). This epidemic requires a multifaceted response, including the delivery of medications and effective lifestyle counseling (3). In low- and middle-income countries, only 36% of people with diabetes receive medication to lower glucose and 19% receive lifestyle counseling (4).

This study investigates the implementation of a diabetes self-management education and support (DSMES) intervention in the public health system in rural Guatemala. Guatemala is the most populous country in the Central America region and has an estimated diabetes prevalence of 9% to 10% (5,6). Diabetes has strained the public health system, which serves more than 70% of the population (7), and has particularly affected rural Indigenous communities (8).

We previously conducted a pilot feasibility study of a culturally tailored, home-based DSMES intervention for Indigenous Maya people (9). DSMES interventions are recommended in Guatemalan primary care guidelines (10) and are effective in ethnic minority groups in high-income countries (11). The pilot intervention used tailored communication theory (12). We subsequently received funding to scale up the DSMES pilot into routine primary public health care centers. The objective of this study was to assess the effectiveness of the DSMES intervention and evaluate this implementation through mixed methods and the RE-AIM framework (13).

## Methods

We prepared this article according to TREND (Transparent Reporting of Evaluations with Nonrandomized Designs) (14) and STARI (Standards for Reporting Implementation Studies) guidelines (15). Checklists are available elsewhere (Appendixes 1 and 2 [16]). This study was approved by the institutional review boards of Maya Health Alliance and the Institute of Nutrition of Central America and Panama.

### Study design and setting

Our DSMES intervention used a pretest–posttest design and was implemented in rural Guatemala from November 2018 through December 2020. The study was conducted by Maya Health Alliance, the Inclusive Health Institute, and the Institute of Nutrition of Central America and Panama. This was a pragmatic study that focused on evaluating DSMES in real-world routine conditions; as a result, we did not perform a sample size calculation.

The study was conducted in 8 rural municipalities in a single province (Chimaltenango) in the Central Highlands region. We chose this province because it is where Maya Health Alliance’s main office is located. The population is predominantly Indigenous Maya (17), and most live below the national poverty line (18). Each municipality has a public health district operated by the Ministry of Health, as well as private and nongovernmental biomedical clinics and nonbiomedical traditional healers. In the public health sector, diabetes care is delivered at a physician-staffed health center. Free services at health centers include blood glucose monitoring and oral glucose-lowering drugs. Patients requiring laboratory assessments, insulin therapy, or specialist management are referred to regional referral hospitals. Delivery of DSMES is limited in this system (8).

### Eligibility and recruitment

We used broad participant inclusion criteria: 1) being aged 18 years or older and 2) having a glycated hemoglobin (HbA_1c_) ≥6.5% or diagnosis of diabetes within the preceding 12 months. We excluded individuals who were pregnant or had type 1 diabetes.

Any health facility in included municipalities was eligible to refer patients. In each of the 8 municipalities, we approached all public health centers and selected public hospitals, private clinics, nongovernmental clinics, and pharmacies. At public health centers, study staff also actively recruited patients from diabetes peer-group meetings. Other recruitment activities included approaching known patients from Maya Health Alliance, word-of-mouth from enrolled participants, door-to-door visits, and public fliers.

### Intervention

The intervention was based on our previous pilot and delivered by community health workers at the participants’ homes (9). The intervention was a public–private partnership whereby community health workers paid by Maya Health Alliance worked within the public health system. The curriculum was adapted for low-literacy Mayan-speaking populations and based on a Guatemalan version of the US National Heart, Lung, and Blood Institute’s Salud Para Su Corazón (Health for Your Heart) model for Latinx populations (19,20). Prior adaptions by our group to this curriculum included diabetes-specific content, home visits with family participation, minimal written text, and culturally relevant drawings, props, and games (9). The intervention consists of a screening visit, 6 monthly education visits, and a closing visit (Box). The curriculum focuses on the “4 pillars” of diabetes control: 1) regular medical appointments, 2) adherence to medications prescribed by health care providers, 3) regular physical activity, and 4) a healthy diet that reduces intake of carbohydrates. At each visit, study educators review achievements from prior visits, assess individualized milestones, and use motivational interviewing to guide participants on overcoming barriers to behavior change. We expected that the total intervention time per participant would be 8 months with each monthly visit lasting 1 hour. We did not provide any incentives to increase participation or adherence.

**Box. Structure and Content of a Diabetes Self-Management Education and Support Intervention in Rural Guatemala, 2018–2020**

**Visit 1**

Screening visit

Baseline data collected

**Visit 2**

“4 Pillars” of type 2 diabetes control: 1) medical visits, 2) medication adherence, 3) diet, and 4) exercise

Normal blood glucose levels

Diabetes symptoms

Diabetes complications

Causes of diabetes

Foot care

**Visit 3**

Diet

Basic food groups

Carbohydrate portions

Activity (does this increase blood glucose?)

Activity (make a healthy plate)

**Visit 4**

Sugary drinks

Snacks

Strategies to eat well at parties

Alcohol consumption

Blood pressure and salt consumption

**Visit 5**

Benefits of exercise

Types of physical activity

Activity (“a day in the life . . .”)

Importance of family support, diet, and exercise

**Visit 6**

Guatemalan beliefs about diabetes

Importance of medications

Importance of medical check

**Visit 7**

Activity (I can control my diabetes!)

Participant-led review activity

Individualized challenges and success (4 pillars of control)

**Visit 8**

Closing visit

End-point data collected

Full intervention materials (facilitator guide and patient visual materials) are available in Spanish at https://doi.org/10.7910/DVN/CUSI4E.

**Impact of COVID-19**. We halted study enrollment in March 2020 when community-based transmission of COVID-19 was reported. Because of safety concerns and mandated travel restrictions, the study transitioned to telephone visits. Participants who had not finished before March 15, 2020, had end-point psychometric data but no end-point clinical data collected.

### Data sources and data collection

We conducted an explanatory sequential mixed-methods evaluation (21) guided by the RE-AIM (Reach, Effectiveness, Adoption, Implementation, Maintenance) Qualitative Evaluation for Systematic Translation (RE-AIM QuEST) mixed-methods framework (13). The project evaluation plan and data sources are available elsewhere (Appendixes 3–4 [16]).

Quantitative data were entered in real time using smartphones and data capture software (REDCap). Quantitative data included participant sociodemographic characteristics, clinical and psychometric outcomes, implementation metrics, and costs. All quantitative data were collected in participants’ homes by 1 trained research assistant. Qualitative data consisted of semistructured interviews with study participants (n = 12), intervention staff (n = 5), and staff members at health centers (n = 6). All intervention staff members and staff members from 6 of 8 health centers were interviewed; 2 health center staff members did not respond. We purposefully sampled interview participants with low and high effect size (change in HbA_1c_) and engagement (average visit time), including at least 1 participant of each sex in each group. The interview guides were designed to explore elements of the quantitative analysis, following dimensions of the RE-AIM framework tailored to each group (13). Example probes included perceptions of project utility, determinants of clinical benefit, impact of COVID-19 and transition to telephone visits, role of sex and other determinants of participation, and family involvement. The full interview guide is available elsewhere (Appendix 5 [16]). Interviews in Spanish lasted approximately 30 minutes. Interviews in Maya Kaqchikel used an interpreter and lasted approximately 1 hour. Interviews were recorded and then translated and transcribed in Spanish. All qualitative data were collected by 1 study author (A.A.), a trained anthropologist, and were conducted via telephone because of COVID-19.

### Outcomes

**Reach.** Reach references the absolute number, proportion, and representativeness of study participants. Quantitatively, we examined lost-to-follow-up and compared the characteristics of study participants with the characteristics of participants with diabetes in a contemporaneous population-representative chronic disease survey conducted by the authors in 2018 and 2019 in one of the study’s municipalities (22). Qualitatively, we focused on barriers to enrollment, especially for men.

**Effectiveness.** Effectiveness references the effect of the intervention on study outcomes. Primary clinical outcomes were HbA_1c_, systolic blood pressure, diastolic blood pressure, and body mass index (BMI). HbA_1c _was assessed with a point-of-care device (A1CNow, PTS Diagnostics). Seated arterial blood pressure was assessed in triplicate after 15 minutes with an Omron 7 digital cuff and estimated as the mean of 3 measurements. Secondary psychometric outcomes were diabetes knowledge, diabetes distress, and self-management. Diabetes knowledge was measured by the Diabetes Knowledge Questionnaire (DKQ-24); scores range from 0 to 24, with higher scores indicating more knowledge (23). Diabetes distress was measured by the Diabetes Distress Scale (DDS); scores range from 1 to 6, with higher scores indicating more distress (24). Self-management was measured by selected questions from the Summary of Diabetes Self-Care Activities instrument (SDSCA) (25). We previously validated the DKQ-24 and SDSCA during our pilot (9). Qualitatively, we investigated the mechanisms influencing effectiveness and potential explanations of differences between participants.

**Adoption.** Adoption references the absolute number, proportion, and representativeness of staff, providers, and organizations. We calculated the proportion of participants who were enrolled by type of health facility (public facilities, private clinics, nongovernmental clinics, pharmacies). We also examined the proportion of participating versus invited facilities. Qualitatively, our interviews explored factors that affected facility participation.

**Implementation.** Implementation references how accurately and consistently the intervention was carried out, including adaptations and cost. We examined total time between screening and closing visits, between first and last education visits, average visit duration, and costs. We also calculated the proportion of the suggested curriculum that was completed during each visit. Qualitatively, we explored barriers to fidelity, strategies to overcome barriers, and intervention modifications.

**Maintenance.** Maintenance references the extent that the intervention and intervention outcomes are sustained after the study. In interviews we explored factors leading to high or low levels of engagement and intent to continue.

### Data analysis

**Quantitative.** We used Stata version 16 (StataCorp LLC) for analyses. We compared participant baseline characteristics with the population-representative sample using the Student *t* test for continuous data and the proportion test for categorical data. Baseline participant characteristics of retained study participants and participants lost to follow-up were also compared using the Student *t* test and proportion test. For clinical outcomes, we constructed multilevel mixed-effects models for HbA_1c_, blood pressure, BMI, the DKQ-24, the DDS, and the SDSCA. Models were prespecified to include random effects for study participant and fixed effects for age, sex, ethnicity, education level, time since diagnosis, difficulty paying for medications, and baseline value.

To investigate the impact of missing data due to COVID-19 and other causes, we conducted a sensitivity analysis using multiple imputation with chained equations and 100 imputations (26). We conducted a second sensitivity analysis of the impact of conducting the intervention virtually during the COVID-19 pandemic on psychometric outcomes.

**Qualitative.** We analyzed interviews using Dedoose (Sociocultural Research Consultants). We conducted a thematic framework analysis using an inductive approach. We first developed a codebook by analyzing 2 interviews from each group. Responses were then coded by 2 authors (S.T. and D.F.), and grouped by RE-AIM dimensions. Differences were resolved through consensus. The final coded interviews added less than 5% new information, indicating thematic saturation (27). 

**Mixed methods.** We integrated our findings using a joint display of quantitative and qualitative findings and meta-inferences (21). Meta-inferences are interpretations that emerge from the integrated analysis of the quantitative and qualitative data. They were generated iteratively by discussion among the team (27).

## Results

Of 738 participants screened, 111 did not meet inclusion criteria. In total, 627 participants were enrolled (Figure). Of all enrolled participants, end-point data were not collected for 23.8% (149 of 627), most of whom had no working telephone number during the COVID-19 lockdown. Psychometric end-point data were collected for 74.6% (468 of 627). Clinical end-point data were collected for 40.0% (251 of 627).

**Figure:**
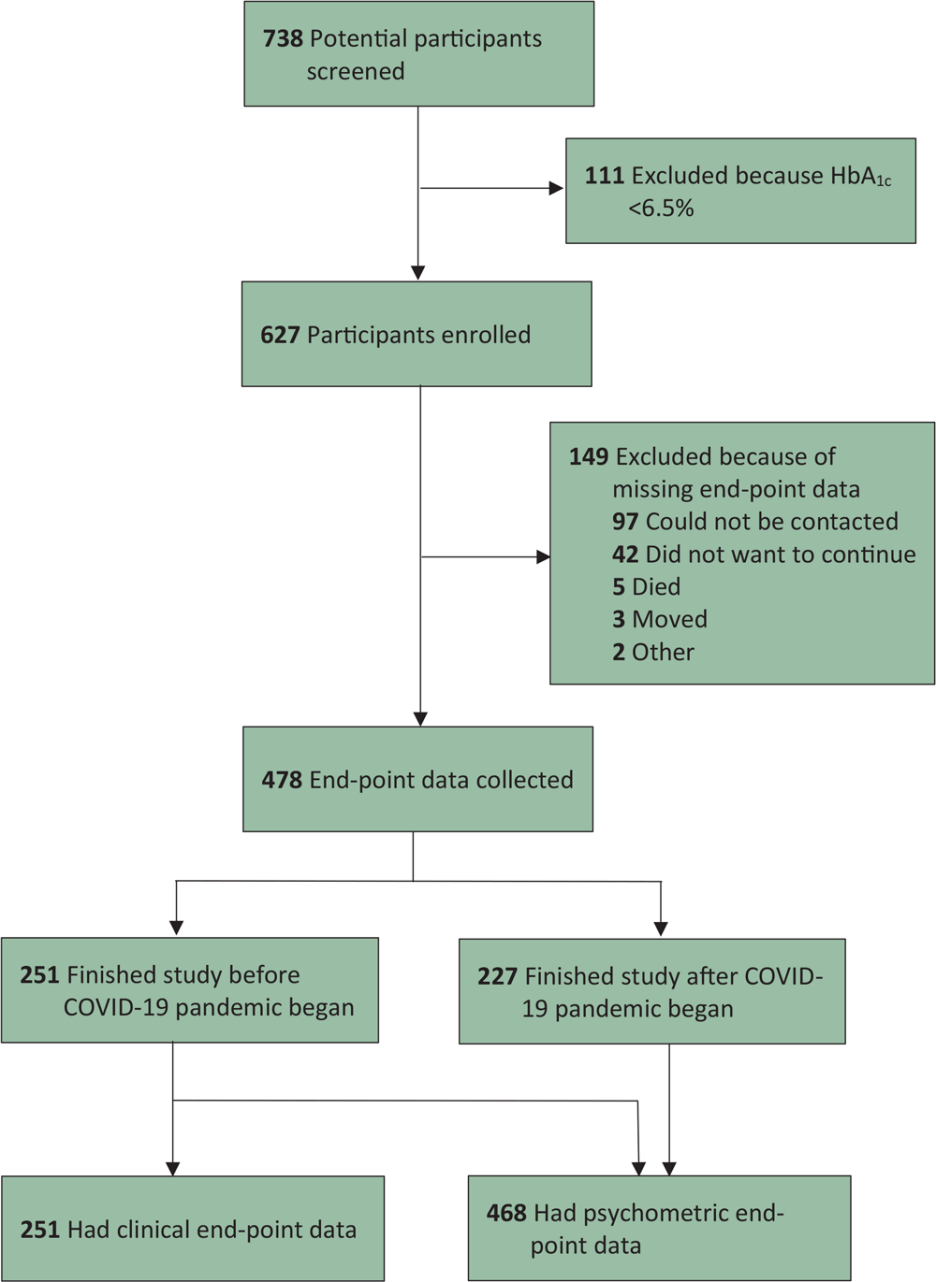
Enrollment flowchart for a diabetes self-management education and support intervention in rural Guatemala, 2018–2020.

### Reach

**Quantitative.** In the comparison of baseline characteristics of participants and the total diabetes population, important differences included the overrepresentation of women, greater preference for a Mayan language, lower levels of education, and higher baseline values for HbA_1c_ and blood pressure (Table 1). Compared with participants who completed the study, participants lost to follow-up were more likely to speak a Mayan language, but otherwise we found no significant baseline sociodemographic or clinical differences, including for self-identified Maya ethnicity. A full comparison of baseline characteristics between participants lost to follow-up and those retained and additional health service data characteristics are available elsewhere (Appendixes 6–7 [16]).

**Table 1:** Baseline Characteristics of Participants Enrolled in a Diabetes Self-Management Education and Support Intervention in Rural Guatemala and Population Comparison, 2018–2020

Variable	Enrolled Participants	Population^a^	*P* Value^b^
Female, %	83.7	47.6	<.001
Age, mean (SD), y	57.3 (12.3)	53.5 (12.4)	.04
Indigenous Maya, %	88.5	71.8	<.001
Preferred language is Mayan, %	56.3	23.1	<.001
Education^c^ is primary or less, %	87.2	50.0	<.001
Years since diagnosis, median (IQR)	7 (3-13)	NA	
**Glycated hemoglobin A_1c_, %**			
Mean (SD)	9.5 (2.1)	8.9 (3.1)	.03
<8.0%	29.7	52.1	<.001
**Blood pressure**			
Systolic, mean (SD), mm Hg	127.8 (21.3)	116.3 (15.9)	<.001
Diastolic, mean (SD), mm Hg	79.9 (10.4)	75.3 (10.7)	.004
Hypertensive, %	28.8	15.5	.052
**Body mass index, kg/m^2^**			
Mean (SD)	28.6 (5.1)	29.3 (5.6)	.37
≥25.0, %	78.0	77.8	.98
≥30.0, %	37.5	35.9	.83

Abbreviations: IQR, interquartile range; NA, not available.

a Individuals with diabetes from a unique population-based survey conducted in the study area during 2018 and 2019 (22).

b Student *t* test for continuous data and proportion test for categorical data.

c Education was treated as a continuous variable in our regression models but is presented in categories here, because only categorical data on education were available in the population survey.

**Qualitative.** In the investigation into reasons for the low levels of enrollment among men, a common theme was that men often leave for work early in the morning and return late, after clinics close. In addition, “*machismo*” (an exaggerated sense of masculinity) negatively affected men’s self-care and their willingness to participate. As one educator reported,

I think it is because of the lack of time men have. The men are the ones who go out to work; they have to go out to the fields to work which doesn’t give them time . . . and I think they are closed in, they don’t like to get checkups often . . . they are embarrassed to say that they have some illness.

Interviewees also reported that many men did not view health education to be of material benefit. One participant shared criticisms of the intervention that he had heard from another person with diabetes:

There is a man who also has diabetes that doesn’t agree with just talks. He said they are only bringing knowledge and lectures. They are not giving medicine or economic support. He said it’s better to invest his time working than talking, then he can buy his own medicine.

### Effectiveness

**Quantitative.** In adjusted multilevel models, mean change in HbA_1c_ was −0.4% (95% CI, −0.6% to −0.3%; *P* < .001), mean change in systolic blood pressure was −5.0 mm Hg (95% CI, −6.4 to −3.7 mm Hg; *P* < .001), mean change in diastolic blood pressure was −2.6 mm Hg (95% CI, −3.4 to −1.9 mm Hg; *P* < .001), and mean change in BMI was 0.5 (95% CI, 0.3 to 0.6; *P* < .001) (Table 2). Mean change in diabetes knowledge assessed using the DKQ-24 was 3.9 (95% CI, 3.6 to 4.1; *P* < .001) and mean change in diabetes distress using the DDS was −0.4 (95% CI, −0.4 to −0.3; *P* < .001). We also found significant improvements in most self-care activities.

**Table 2:** Primary and Secondary Outcomes of Participants in a Diabetes Self-Management Education and Support Intervention in Rural Guatemala, 2018–2020^a^

Outcome	Baseline	End Point	Adjusted Pre-Post Difference, Mean Change (95% CI)	*P* Value^b^
**Primary outcomes, mean (SD)**				
Glycated hemoglobin A_1c_, %	9.5 (2.1)	8.9 (2.0)	−0.4 (−0.6 to −0.3)	<.001
Systolic blood pressure, mm Hg	127.8 (21.3)	123.2 (19.5)	−5.0 (−6.4 to −3.7)	<.001
Diastolic blood pressure, mm Hg	79.9 (10.4)	76.9 (10.1)	−2.6 (−3.4 to −1.9)	<.001
Body mass index, kg/m^2^	28.6 (5.1)	28.6 (4.7)	0.5 (0.3 to 0.6)	<.001
**Secondary outcomes, mean (SD)**				
Diabetes Knowledge Questionnaire-24^c^	12.0 (3.9)	16.2 (2.9)	3.9 (3.6 to 4.1)	<.001
Diabetes Distress Scale^d^	2.5 (0.8)	2.1 (0.7)	−0.4 (−0.4 to −0.3)	<.001

**Summary of Diabetes Self-Care Activities^e^**				
**Median (IQR) number of days in the last week you have . . .**				
Followed a healthy diet	3 (1-4)	4 (3-5)	2.1 (1.8 to 2.3)	<.001
Exercised ≥30 min	1 (0-3)	2 (1-3)	1.2 (1.0 to 1.5)	<.001
Checked feet	2 (0-4)	3 (2-7)	1.5 (1.3 to 1.8)	<.001
Taken medications	6 (4-7)	6 (5-7)	0.2 (0 to 0.5)	.10
**Answered yes to yes/no question**				
Know what a carbohydrate is	0.06 (0.05 to 0.08)	0.39 (0.35 to 0.44)	0.32 (0.28 to 0.36)^f^	<.001
Smoked in the last week	0.03 (0.02 to 0.04)	0.01 (0.00 to 0.03)	−0.02 (−0.02 to −0.01)^f^	.001

Abbreviation: IQR, interquartile range.

a All models were hierarchical mixed-effect models that included a random-intercepts effect for study participant. Adjusted models included fixed effects for the intervention time, age, sex, ethnicity, education level, time since diagnosis, difficulty paying for medications, and baseline value. Primary and secondary outcomes were linear regression models. Days per week in self-care activities were assessed in ordinal regression models, and yes/no questions in logistic regression models, where 0 = no and 1 = yes.

b Determined by linear mixed-effects model.

c Scores range from 0 to 24, with higher scores indicating more knowledge (23).

d Scores range from 1 to 6, with higher scores indicating more distress (24).

e Self-management was measured by selected questions from the Summary of Diabetes Self-Care Activities instrument (25).

f Values are marginal effect (95% CI).

The results from the sensitivity analysis investigating the impact of missing data through multiple imputation were similar to the results of the primary analysis; details are available elsewhere (Appendix 8 [16]). All missing data for this exercise were imputed, except for binary SDSCA outcomes because of multicollinearity. In the second sensitivity analysis, which assessed the impact of conducting the intervention virtually during the COVID-19 pandemic, improvements in knowledge, distress, and diet outcomes were similar before and during the pandemic; details are available elsewhere (Appendix 9 [16]). However, we did not observe improvements in physical activity outcomes during the pandemic.

**Qualitative.** The primary mechanism affecting effectiveness was the personalized nature of visits, which addressed participants’ specific needs while building trust. Themes mentioned included 1) the tailoring of educational content to each participant, 2) the supportiveness of educators, 3) the favorability of home-based visits, and 4) the patients’ ability to choose a preferred language. As 1 study educator explained,

If the patient preferred to speak in Kaqchikel, I would speak to them in Kaqchikel; if they wanted to speak in Spanish, then I would speak Spanish. I think it is very important that participants receive the education in their preferred language. This gives them more confidence . . . it is much better to have personalized education because the participant can express their doubts and not be embarrassed or worried about what their peers hear . . . in our program we go step by step, theme by theme, personalized to the participant.

Another educator commented on the role of family and community support:

When the family participated it had a great influence on the participant. When the family attended the education visits and understood it, they could support each other at home and throughout the following days. When there was family support, there was more positive changes in the participants.

### Adoption

**Quantitative.** Of the 612 participants, 386 (63.1%) were referred from health centers; 31.4% (192 of 612) from Maya Health Alliance programs; 4.2% (26 of 612) from private clinics; 1.0% (6 of 612) from the regional public hospital; and 0.3% (2 of 612) through door-to-door promotion.

All 44 health facilities approached agreed to participate. Of 10 public health facilities, 8 were health centers and 2 were hospitals. Seven of 8 health centers and 1 of 2 public hospitals referred patients. Of 24 private clinics, only 3 referred patients. None of the 8 pharmacies who agreed to participate referred participants.

**Qualitative.** Interviews highlighted partial adoption by participating health centers. Health center staff allowed recruitment of participants attending diabetes peer groups but were otherwise not active. One study educator acknowledged this lack of integration: “All we did was present the project to the directors to get approval. They gave us 10 to 15 minutes to present our project at the diabetes club meetings and that was it.”

One identified barrier that prevented adoption was the lack of a training program for health center staff. One study educator noted this could be improved: “In the future we could coordinate with the health centers to do trainings with the staff working in diabetes. We could train the staff and also the health center directors before the intervention so that they are more involved.”

### Implementation

**Quantitative.** The median time between baseline and end-point data collection was 268 (interquartile range, 225–343) days. The median time between first and last education visits was 155 (interquartile range, 144–182) days. During the intervention (pre– and post–COVID-19), 83.7% (525 of 627) of participants completed all 6 education visits. The mean (SD) duration of home visits was 70.9 (15.4) minutes. The mean (SD) duration of telephone visits was 41.4 (13.2) minutes.

Direct intervention costs were US$90.19 per participant (Appendix 10 [16]). In comparison, government health expenditure per capita is US$94.49, and total current health expenditure per capita is US$259.62, with 57.5% of costs being out of pocket (28).

The median of suggested curriculum elements that were completed for all visits was 94.3% (interquartile range, 91.8%–96.5%).

**Qualitative.** The main intervention modifications were caused by the COVID-19 pandemic. Implementing the intervention was more difficult after the transition to virtual visits. One positive aspect was the ability to schedule visits during nonworking hours. One study educator summarized:

One challenge was that cellular reception was very bad . . . I had to call 3 to 4 times to finish a study visit. Also, the phone numbers we had were often not the participants’. . . . When we called, the participant would not be home, and it was uncomfortable for the family member or neighbor. This gave us less time for the visit. Phone visits had fewer questions than home visits because we could not show them pictures, which helped generate a lot of questions. . . . We did cover all the topics, but the patients were a little more closed.

Although home visits were preferred, participants were generally satisfied with the quality of telephone visits: “Phone visits are fine. It is not that I don’t like them, they were fine and logical, but if it is possible home visits are better.”

### Maintenance

**Qualitative.** All interview participants desired to continue practicing what they had learned during the intervention. A common theme was that changes were difficult but became easier over time: “[T]he beginning was the most difficult because humans are used to eating what they want to. You have no diet, you eat everything. But later you start adapting to the diet and eventually you are used to it and it is easier.”

At the organizational level, all health center staff expressed support. The main barrier to continuing the intervention was lack of time: “I think that our availability, our time would be the biggest challenge. I don’t think that the intervention would be difficult for us to do, but the time we have is what would be difficult.”

### Mixed methods

We summarized the quantitative and qualitative findings and meta-inferences globally because we found no significant differences between the purposefully sampled groups (Table 3). First, DSMES interventions struggle to enroll men when they lack strategies that accommodate work schedules and address cultural barriers to self-care and education. Second, although all types of health facilities were eager to participate in identifying and referring participants, their day-to-day participation was limited. Better integration is critical in scaling up DSMES interventions in the public health system. Third, although the public health center system has interest in “personalized” DSMES, achieving sustainability requires addressing budgetary and time constraints. A key sustainability limitation of the public–private approach used here was that the public sector’s capacity to adequately support the cost of DSMES staff was unclear.

**Table 3:** Explanatory Sequential Joint Display: A Summary of Quantitative and Qualitative Findings and Mixed Methods Meta-Inferences in an Evaluation of a Diabetes Self-Management Education and Support Intervention in Rural Guatemala, 2018–2020

RE-AIM Dimension	Quantitative Findings	Qualitative Findings	Meta-Inferences
**Reach**	• 16% of participants were men, while approximately 50% of people with diabetes in the population were men • Participants had worse HbA_1c_ and blood pressure compared with overall diabetes population	Barriers to enrollment of men: • Prioritization of work • Culture of machismo • DSMES not perceived as beneficial • Desire to received something of material value for time (also found for women)	Future DSMES interventions may have trouble reaching total diabetes population without • Prioritizing at-work men • Addressing the culture of machismo • Integrating education more clearly within the broader structures of clinical diabetes care
**Effectiveness**	Improvements in clinical and psychometric outcomes: • HbA_1c_, blood pressure • Diabetes knowledge, diabetes distress, self-care activities	Principal mechanisms that led to effectiveness: • Personalized nature of study visits • Cultural and linguistic acceptability • Family and community support	DSMES programs benefit from: • Patient-centered care • Family and community inclusion
**Adoption**	• Most (95%) participants were recruited from health centers or by Wuqu’ Kawoq staff and programs • All health facilities that were approached agreed to participate, although few patients were referred	• Intervention was only partially adopted by health centers • Pre-intervention trainings may help increase health facility involvement	• Public and private health facilities were willing to participate in the DSMES program • Minimal participation in settings without direct involvement of study staff • Special attention to integrating health facilities may be necessary
**Implementation**	Mean visit duration: • Home visits (71 min) • Telephone visits (41 min)	• More difficult to implement telephone visits than home visits • Overall high levels of patient satisfaction with telephone visits	Future interventions should carefully consider tradeoffs between at-home and telephone visits
**Maintenance**	Direct intervention costs were US$90.19 per participant	Both participants and health center staff expressed desire to continue the intervention	There is interest in sustaining DSMES from: • Patients • Health workers • Health facility leadership However, important financial and time constraints exist

Abbreviations: DSMES, diabetes self-management education and support; HbA_1c_, glycated hemoglobin A_1c_; RE-AIM, reach, effectiveness, adoption, implementation, and maintenance.

## Discussion

This study reports outcomes of a culturally tailored DSMES intervention scaled up within the public health system in rural Guatemala. The intervention led to substantial improvements in clinical and psychometric outcomes despite challenges posed by the COVID-19 pandemic.

A recent systematic review of DSMES interventions in low- and middle-income countries concluded that evidence is limited by study heterogeneity and that randomized controlled trials are needed (29). However, it is unlikely that randomized controlled trials will be completed in most settings where diabetes is a pressing concern, not just because of cost but also because of 1) the face validity of DSMES principles, and 2) their recognition as standard of care in high-income settings (9,30). Therefore, analyses of nonexperimental interventions using detailed implementation assessment frameworks can provide data to assist policy makers.

Our study is the largest observational study on type 2 diabetes in Guatemala, and several findings are worth highlighting. First, access to medical care and medications at baseline was higher than previously reported during the last decade (Table 2, Appendix 7 [16]), likely a result of efforts by the Ministry of Health’s Chronic Disease Commission to strengthen chronic disease care in rural centers (9). On the other hand, baseline self-care indicators for diet and exercise were low, suggesting that education has not kept pace. A recent pooled analysis emphasizes that lifestyle education for diabetes is a major unmet need globally (30).

Our analysis of barriers and facilitators to implementation highlights several important challenges to scaling DSMES in low-resource settings. First, although men commonly are underreached by these initiatives (30), limited data on disease prevalence has made precise assessments difficult. We found that women were overrepresented by nearly 40 percentage points. Second, participant interviews highlighted that the high degree of personalization of the intervention was essential for their engagement. This personalization likely fosters effectiveness by building a trusted supportive relationship. This is an important point for future scalability, as health center staff members felt that time and budgetary constraints would make precisely this degree of personalization infeasible. Solving these staffing constraints on tailored interactions with patients is critical to scaling lifestyle interventions in low-resource settings (12). In particular, it is likely that dedicated DSMES providers — analogous to certified diabetes educators — are essential to effective relationship building, and future work will need to explore this as an alternative to the more typical “generalist” frontline worker model common in Guatemala and the region.

Our study has several limitations and strengths. First, our study focuses on DSMES in one geographic area of Guatemala, although this is balanced by strengths such as the pragmatic design, the large sample size, and the comparison to a representative population sample permitting assessment of reach. Second, we used a pretest–posttest study design, which limits our ability to report causality, although we did adjust the analysis for important prespecified covariates. Third, the intervention was limited to a duration of 6 months. Planned 12-month assessments were abandoned because of the COVID-19 pandemic. Fourth, because of COVID-19, we modified our intervention and evaluation plan, resulting in missing outcome data. We addressed these issues through sensitivity analyses, including multiple imputation (Appendixes 8–9 [16]). The findings from these sensitivity analyses support our primary conclusions.

We found that in a rural population of individuals with type 2 diabetes in Guatemala, a community health worker–led DSMES intervention within the public health system led to improvements in HbA_1c_, blood pressure, diabetes knowledge, disease-related stress, and diet and physical activity. Our mixed-methods implementation research shows that scaling up DSMES in low-resource health systems requires careful consideration of implementation barriers and facilitators. Long-term staffing and cost of the intervention are also important concerns.
